# The Prevalence of Gastrointestinal Bleeding in COVID-19 Patients: A Systematic Review and Meta-Analysis

**DOI:** 10.3390/medicina59081500

**Published:** 2023-08-21

**Authors:** Eleni Karlafti, Dimitrios Tsavdaris, Evangelia Kotzakioulafi, Adonis A. Protopapas, Georgia Kaiafa, Smaro Netta, Christos Savopoulos, Antonios Michalopoulos, Daniel Paramythiotis

**Affiliations:** 1 Emergency Department, University General Hospital of Thessaloniki AHEPA, Aristotle University of Thessaloniki, 54636 Thessaloniki, Greece; 2 1st Propaedeutic Department of Internal Medicine, AHEPA University General Hospital, Aristotle University of Thessaloniki, 54636 Thessaloniki, Greece; evelinakotzak@hotmail.com (E.K.); adoprot@hotmail.com (A.A.P.); gdkaiafa@auth.gr (G.K.); chrisavopoulos@gmail.com (C.S.); 3 1st Propaedeutic Surgery Department, University General Hospital of Thessaloniki AHEPA, 54636 Thessaloniki, Greece; tsavdaris@auth.gr (D.T.); smaronetta2@gmail.com (S.N.); amichal@auth.gr (A.M.); danosprx@auth.gr (D.P.)

**Keywords:** COVID-19, gastrointestinal bleeding

## Abstract

*Introduction*: Severe acute respiratory syndrome coronavirus 2 caused the coronavirus disease of 2019 (COVID-19), which rapidly became a pandemic, claiming millions of lives. Apart from the main manifestations of this infection concerning the respiratory tract, such as pneumonia, there are also many manifestations from the gastrointestinal tract. Of these, bleeding from the gastrointestinal tract is a significant complication quite dangerous for life. This bleeding is divided into upper and lower, and the primary pathophysiological mechanism is the entering of the virus into the host cells through the Angiotensin-converting enzyme 2 receptors. Also, other comorbidities and the medication of corticosteroids and anticoagulants are considered to favor the occurrence of gastrointestinal bleeding (GIB). *Methods*: This systematic review was conducted following the Preferred Reporting Items for Systematic Reviews and Meta-Analyses (PRISMA) guidelines, and the studies were searched in two different databases (Scopus and PubMed) from November 2019 until February 2023. All studies that reported GIB events among COVID-19 patients were included. *Results*: 33 studies were selected and reviewed to estimate the prevalence of GIB. A total of 134,905 patients with COVID-19 were included in these studies, and there were 1458 episodes of GIB. The prevalence of GIB, in these 33 studies, ranges from 0.47% to 19%. This range of prevalence is justified by the characteristics of the COVID-19 patients. These characteristics are the severity of COVID-19, anticoagulant and other drug treatments, the selection of only patients with gastrointestinal manifestations, etc. The pooled prevalence of gastrointestinal bleeding was estimated to be 3.05%, rising to 6.2% when only anticoagulant patients were included. *Conclusions*: GIB in COVID-19 patients is not a rare finding, and its appropriate and immediate treatment is necessary as it can be life-threatening. The most common clinical findings are melena and hematemesis, which characterize upper GIB. Treatment can be conservative; however, endoscopic management of bleeding with embolization is deemed necessary in some cases.

## 1. Introduction

Severe acute respiratory syndrome coronavirus 2 (SARS-CoV-2) is a virus that belongs to the Coronaviridae family [1]. It is a highly contagious virus, so Coronavirus disease 2019 (COVID-19), which started in Wuhan, China in December 2019, rapidly spread into a pandemic [2,3]. It is a pandemic that counts millions of deaths and more than 700 million cases. Fortunately, most cases do not require hospitalization. The virus is transmitted through aerosols and droplets, so it can easily be transmitted [2,4]. Its diagnosis can be achieved in several ways [5]. The reverse transcription polymerase chain reaction (rt-PCR) test is the gold standard technique for the diagnosis of SARS-CoV-2 [5,6], while other methods include rapid antigen SARS-CoV-2 tests [7,8]. The best way to deal with the pandemic is prevention, specifically vaccination of the population, the effectiveness of which can exceed 85% [9].

As for the symptoms of COVID-19, they appear on average 5 to 7 days after infection [3]. The most common of these are fever, fatigue, cough, and respiratory system symptoms [2,3]. However, quite often gastrointestinal symptoms also appear [2]. These include diarrhea, nausea, vomiting, anorexia, and abdominal pain [4,10]. The mechanism for GI involvement in COVID-19 is explained by the expression of angiotensin-converting enzyme 2 (ACE2) receptors in the gastrointestinal system. SARS-CoV-2 can bind to these receptors and enter host cells through the spike protein on its surface. The ability of the virus to escape from the body’s immune system and the activation of the Furin protease before entering the host cell also contribute to this mechanism [2,4,10,11,12]. Another gastrointestinal symptom in patients suffering from COVID-19 is gastrointestinal bleeding [10,11].

Gastrointestinal bleeding (GIB) is distinguished into upper and lower bleeding with a characteristic border of the Treitz ligament. Upper gastrointestinal bleeding (UGIB) is defined as the loss of blood above the ligament of Treitz, i.e., into the duodenum, stomach, and esophagus. Its most common manifestations are melena and hematemesis [13,14]. The severity of UGIB depends on the patient’s hemodynamic status, which also determines how to treat the bleeding [14]. In contrast, lower gastrointestinal bleeding (LGIB) is defined as bleeding below the ligament of Treitz [15,16]. These two types of bleeding (UGIB and LGIB) are different clinical entities and are treated differently. However, both can be a complication of COVID-19 [10].

It has not been ascertained whether the gastrointestinal bleeding in COVID-19 patients is due to the disease itself; however, it seems that the bleeding is due to other causes (e.g., perforation of a peptic ulcer or hemorrhagic gastritis) and that the pathophysiological mechanism of the COVID-19 plays a secondary role. This pathophysiological mechanism of GIB in patients with COVID-19 is explained by the entrance of SARS-CoV-2 into intestinal host cells through ACE-2 receptors that are highly expressed in gastrointestinal organs [10,11]. This entrance causes a local gastrointestinal infection that can lead to bleeding [17,18]. Bleeding can also be favored by medication against the COVID-19 infection, which usually consists of corticosteroids and anticoagulants (to treat the hypercoagulability that COVID-19 infection can cause) [19]. Finally, sepsis, pneumonia, and multiple organ failure may be causes of GIB in severely ill patients with COVID-19 [20]. This systematic review and meta-analysis aimed to determine the prevalence of GIB in patients suffering from COVID-19.

## 2. Materials and Methods

### 2.1. Study Protocol and Guidelines

This systematic review was conducted according to the Preferred Reporting Items for Systematic Reviews and Meta-Analyses (PRISMA) guidelines [21] and the MOOSE (Meta-analysis of observational studies in Εpidemiology) guidelines [22]. This systematic review is registered in the Open Science Framework (OSF) with registration number: 10.17605/OSF.IO/M7PAF.

### 2.2. Eligibility Criteria

Eligibility criteria for this systematic review are presented in Table 1 and were defined with the PICO framework, which is the most-used model for structuring clinical questions. Observational cohort studies and cross-sectional studies with COVID-19 patients reporting GI bleeding events or prevalence of GIB were eligible for inclusion. Systematic reviews, case reports, letters to the editor, and conference abstracts were excluded.

### 2.3. Search Strategy and Selection Process

In February 2023, the systematic literature search was conducted in two databases (PubMed and Scopus). The keywords used were ‘COVID-19’ and ‘Gastrointestinal Bleeding’. The time limit was from November 2019 (emergence of SARS-CoV-2 in China) until February 2023. The selection process was carried out by two independent reviewers (D.T. and E. Kar.) who assessed the studies’ eligibility for inclusion in this systematic review using the title, abstract, and full-text evaluation in the Rayyan platform for systematic reviews. Any disagreement was solved by a third reviewer (E. Kot.).

### 2.4. Data Collection Process and Data Items

Data extraction was carried out by one reviewer (D.T.), and another reviewer (E. Kot.) independently checked the results. The data were extracted in a standardized Excel form. Data extraction included study details, like the author, year, country, total participants, GI bleeding prevalence, GI bleeding events, mean age of participants, and gender if mentioned. The main characteristics of each study are presented in Table 2.

### 2.5. Quality Assessment

Quality assessment of the included studies was performed using the Newcastle-Ottawa Quality Assessment Scale for Cohort Studies by two independent reviewers (D.T., E. Kar.). Any disagreement was solved by a third reviewer (E. Kot.). The Newcastle-Ottawa tool consists of three evaluation domains. These are the selection of participating patients, comparability, and outcomes.

Selection of participating patients includes Representativeness of the exposed cohort, Selection of the non-exposed cohort, Ascertainment of exposure, and Demonstration that the outcome of interest was not present at the start of the study. The first question regarding the representativeness of the exposed cohort is given a star when it comes to Truly or Somewhat representative. Regarding the Selection of the non-exposed cohort, a star is given if the Selection of the non-exposed cohort is drawn from the same community as the exposed cohort. To be given a star in the field Ascertainment of exposure, it must be Secure record or Structured interviews. Finally, the last star is given if there is a Demonstration that the outcome of interest was not present at the start of the study.

The 2nd domain on comparability gives two stars related to the comparability of cohorts based on design or analysis that controlled for confounders.

For the outcomes, the Assessment of the outcome and the follow-up are evaluated. For the first, a star is given if it is an Independent blind assessment or Record linkage, while, for the follow-up if it was sufficient to evaluate the results and if several of the participants submitted to it.

### 2.6. Statistical Analysis

Statistical analysis was performed with RevMan Software by Cochrane (v.5.3). Meta-analysis of the GI bleeding prevalence was performed using a random effects model. Heterogeneity between studies was evaluated with the I^2^ and Q statistic. The investigation for publication bias was performed with the construction of funnel plots.

The statistical analysis was performed by one reviewer (D.T.), and another reviewer (E. Kot.) independently checked the results.

## 3. Results

### 3.1. Study Selection

**Figure 1:**
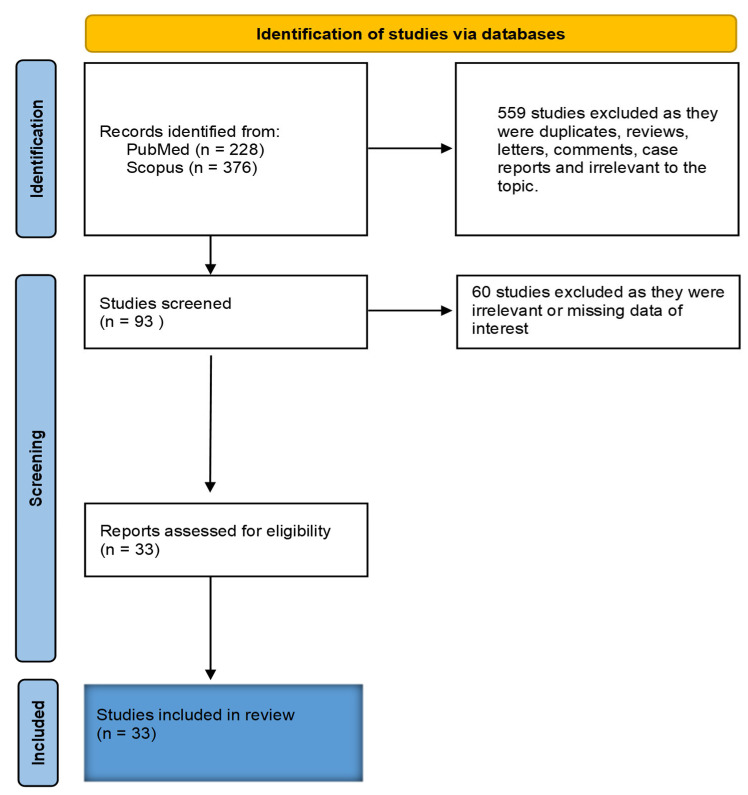
PRISMA flow chart summarising study identification and selection.

A total of 604 studies were identified from databases PubMed and Scopus after searching the keywords ‘COVID-19 patients’ and ‘Gastrointestinal Bleeding’. From these studies, 559 were excluded as duplicates, reviews, letters, case reports, and comments. Thus, the 93 remaining studies were screened. A total of 60 of these studies did not meet the inclusion criteria of this systematic review and were therefore excluded. Thus, 33 studies were assessed for eligibility. All 33 were included in this systematic review to estimate the prevalence of GIB in COVID-19 patients. The identification of studies for this systematic review is illustrated in the PRISMA 2020 flow chart (Figure 1). All selection stages from the initial stage to the final stage are depicted [23,24].

### 3.2. Study Characteristics

Among the 134,905 COVID-19 patients who participated in these 33 studies, 1458 GIB cases were reported. Of these 134,905 COVID-19 patients, the majority are male, while, at the same time, the median age exceeds 40 in all 33 studies. These studies took place in 13 different countries (China, USA, Russia, India, Brazil, Germany, Spain, Italy, Denmark, Finland, Kuwait, Romania, and Turkey). The largest study was carried out in Spain and involved 74,814 COVID-19 patients in 62 Spanish EDs. The prevalence of GIB in COVID-19 patients varies among the 33 studies and ranges from 0.47% to 19%. This large difference mainly depends on the characteristics of the COVID-19 patients who participated in the studies, which are related to taking anticoagulants or the existence of comorbidities. The characteristics of each study are presented in Table 2. 

**Table 2:** Study Characteristics of included studies.

Study ID	Location	Total Subjects (% Male/Median Age)	Study Type	Gastrointestinal Bleeding Cases (Prevalence %)
Chen et al., 2021 [25]	China	2552 (50.4/57.8)	ORCS	40 (1.6)
Alakuş et al., 2022 [26]	Turkey	5484 (73/70.1)	ORCS	44 (0.8)
Mauro et al., 2021 [27]	Italy	4871 (78.3/75)	ORCS	23 (0.47)
Trindade et al., 2021 [28]	USA	11,158	CCS	314 (3)
Makker et al., 2021 [29]	Finland	1206 (60.8/62)	ORCS	37 (3.1)
Popa et al., 2022 [30]	Romania	1881 (66.6%)	ORCS	11 (0.58)
Prasoppokakorn et al., 2022 [31]	Thailand	6373 (65.1/69.1)	ORCS	43 (0.7)
González et al., 2022 [32]	Spain	74,814	ORCS	83 (1.11)
Lak et al., 2022 [33]	China	381 (61.4/62.6)	CS	16 (4.2)
Rosevics et al., 2021 [34]	Brazil	631(54.2/56.7)	CS	10 (1.6)
Zellmer et al., 2021 [35]	NM	5344 (57.1%)	SR	97 (1.8)
Abowali et al., 2022 [36]	USA	651 (54.2/66)	ORCS	16 (2.85)
Abulawi et al., 2022 [37]	USA	1007 (56/63)	CCS	76 (8)
Shalimar et al., 2021 [38]	India	1342 (70.8/45.8)	ORCS	24 (1.8)
Lin et al., 2020 [39]	China	95 (47/45.3)	ORCS	6 (6.3)
Shao et al., 2020 [40]	China	18 (72.2/73.5)	ORCS	1 (5.6)
Fanning et al., 2023 [41]	NM	11,969	ORCS	276 (2.3)
Zhao et al., 2021 [42]	China	368 (51.7/59)	ORCS	43 (11.7)
Martin et al., 2020 [43]	USA	987	CCS	41 (4.15)
Xiao et al., 2020 [44]	China	73 (56.1/43)	ORCS	10 (13.7)
Al-Samkari et al., 2020 [45]	USA	400	ORCS	19 (4.8)
Wan et al., 2020 [46]	China	232 (56/47)	ORCS	10 (4)
Mattioli et al., 2021 [47]	Italy	105 (58/73.7)	ORCS	2 (1.9)
Patell et al., 2020 [48]	USA	398 (52.5%)	ORCS	33 (8.29)
Bunch et al., 2021 [49]	USA	79 (65.8/71)	ORCS	2 (2.81)
Qiu et al., 2021 [50]	China	34 (71/66)	ORCS	6 (17.6)
Russell et al., 2022 [51]	Denmark	1377 (68/68)	ORCS	108 (8)
Bychinin et al., 2022 [52]	Russia	442 (43.5/78)	ORCS	9 (2)
Bonafni et al., 2022 [53]	Italy	30 (63/68.5)	ORCS	3 (10)
Abdelmohsen et al., 2021 [54]	Kuwait	30 (70/57.7)	ORCS	6 (20)
Neuberger et al., 2022 [55]	Germany	51	ORCS	2 (3.90)
Nikolay N. et al., 2022 [56]	Russia	387 (29.9/65.4)	ORCS	22 (5.7)
Demelo-Rodriguez et al., 2021 [57]	Spain	132 (47%)	ORCS	25 (19)

ORCS: Observational retrospective cohort study, CS: cross-sectional study, CCS: case-control study, SR: Survey Research.

### 3.3. Clinical Findings

**Figure 2:**
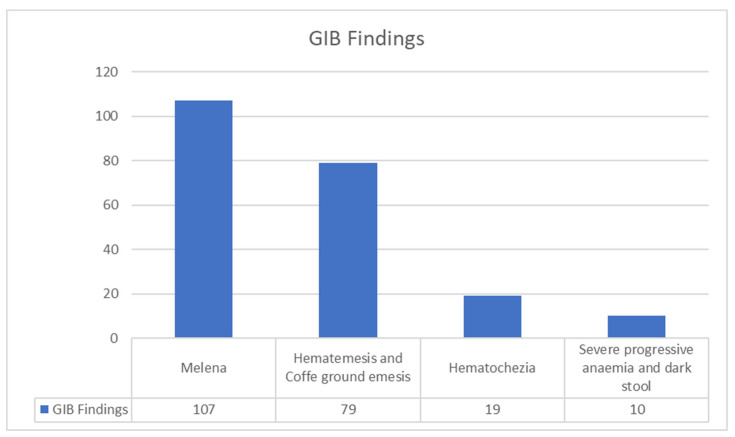
Clinical findings of patients with GIB. For the rest of the patients, the clinical findings are not reported.

Among the 1458 cases of GIB, 107 were manifested by melena, 79 by hematemesis and coffee ground emesis, 19 by haematochezia, 10 by severe progressive anemia and dark stool, while for the remaining cases, their manifestation and their clinical findings were not mentioned. Some of these clinical findings, such as hematemesis and melena, may have coexisted. These clinical findings are also illustrated in Figure 2.

It does not include all patients with GIB but only those clinical findings that were reported in the 33 studies. Hematemesis and melena indicate UGIB.

#### 3.3.1. Comorbidities

**Figure 3:**
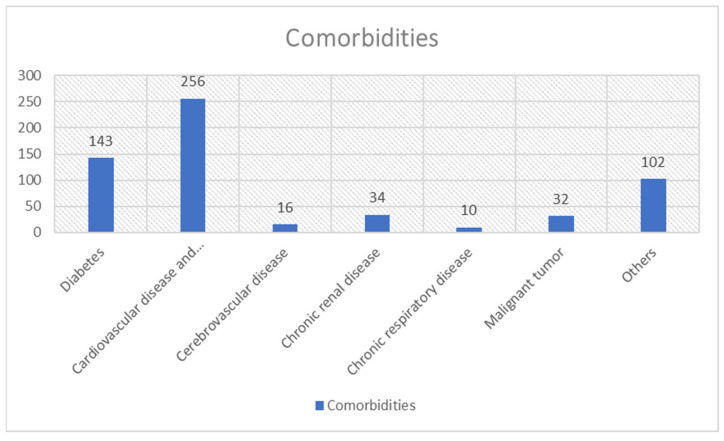
The main comorbidities of COVID-19 patients who experienced gastrointestinal bleeding.

Comorbidities of patients with GIB and COVID-19 were also reported. These mostly concern cardiovascular diseases and hypertension (256/1458) and diabetes (143/1458). Less common were comorbidities involving renal disease (34/1458) and malignant tumors (32/1458). Cerebrovascular disease (16/1458) and chronic respiratory disease (10/1458) were rare. Finally, various other comorbidities were reported (102/1458) such as cirrhosis and liver diseases. These comorbidities are shown in Figure 3.

Many of the above-mentioned comorbidities may have coexisted.

#### 3.3.2. Endoscopy Characteristics

**Figure 4:**
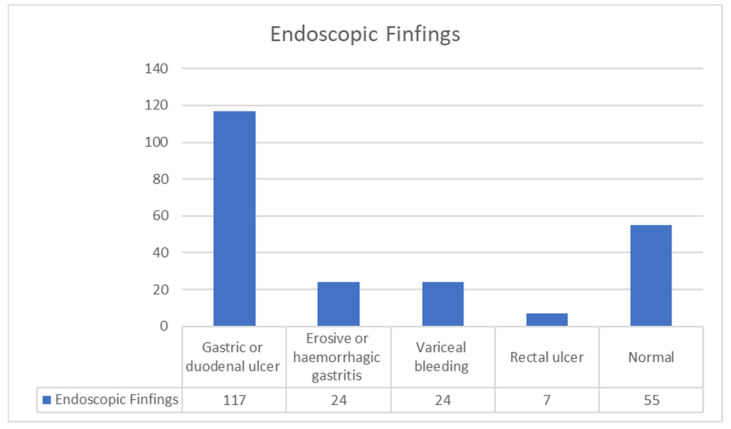
Findings of endoscopic procedures.

Of these 1458 cases, 286 underwent endoscopic procedures. The endoscopic findings show that the most common finding of GIB in COVID-19 patients is a gastric or duodenal ulcer (117 patients). Erosive or hemorrhagic gastritis was found in 24 patients, and variceal bleeding was also found in 24 patients. A rectal ulcer is a rarer finding and was found in 7 patients. In 55 patients, no pathological finding was found in endoscopies. The distribution of the findings is shown in Figure 4.

#### 3.3.3. Laboratory Findings

A total of 19 of the 33 studies [26,27,28,29,31,32,35,36,37,38,40,42,43,45,47,51,52,53,57] included in this systematic review also report the laboratory findings of patients with COVID-19 who developed gastrointestinal bleeding. The Table 3 below shows these laboratory findings which are specifically hematocrit, hemoglobin, platelets, prothrombin time, INR, and d-dimers [58,59,60,61].

#### 3.3.4. COVID-19 Treatments during Hospitalization in Patients with GIB

The studies in this systematic review refer to the administration of corticosteroids, anticoagulants, and proton pump inhibitors. Specifically, 112 patients received corticosteroids (administered due to hyperinflammation [62] and acute respiratory distress syndrome [63,64,65]). A total of 215 received anticoagulation due to coagulation disorders [66,67,68,69,70,71]. A total of 110 received low molecular weight heparin. Finally, 141 received proton pump inhibitors (to prevent bleeding in COVID-19 patients) [72,73]. These results are shown in Table 4 and concern only the incidents reported in the studies and not all patients.

#### 3.3.5. Outcomes

**Figure 5:**
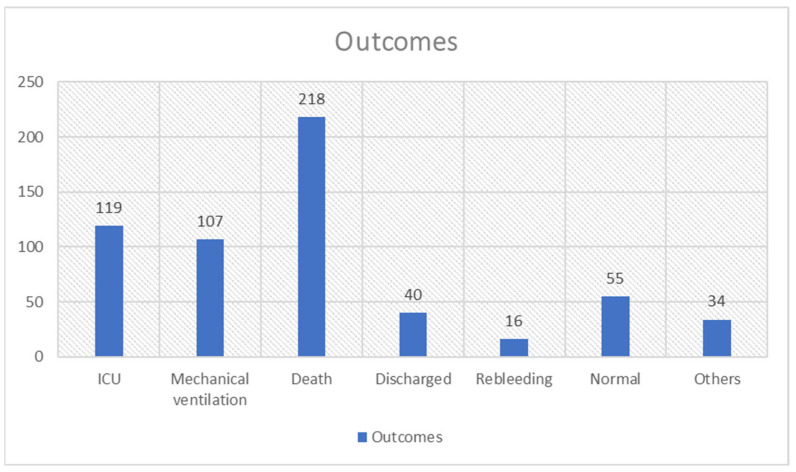
Outcomes of COVID-19 patients with GIB.

Regarding outcomes, 119 patients were admitted to the intensive care unit, while 107 COVID-19 patients were intubated with mechanical ventilation. A total of 40 patients were discharged from the hospital, while 218 patients died. Finally, 16 rebleeding patients were reported. Outcomes are presented in Figure 5. These deaths were not solely due to gastrointestinal bleeding but to the general aggravated condition of the patients and the co-complications of the disease such as respiratory failure.

### 3.4. Quality Assessment

Quality assessment of the included studies was performed using the Newcastle-Ottawa Quality Assessment Scale for Cohort Studies by two independent reviewers (D.T., E. Kar.). The results are listed in Table 5. In the studies in our systematic review, the majority did not use a non-exposed cohort for this and were not given a star in the selection domain. All studies were evaluated with good methodological quality except for Shao et al., 2020 [40] which was considered fair quality.

### 3.5. Meta-Analysis Results

**Figure 6:**
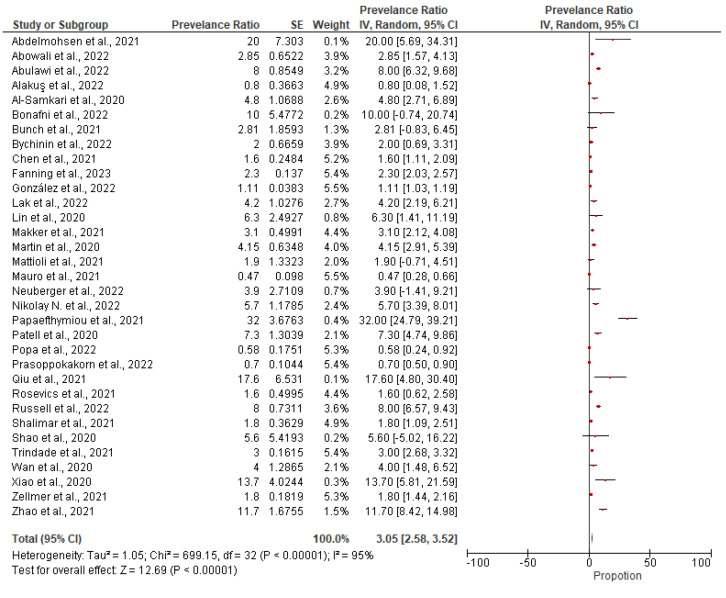
Forest plot of the pooled prevalence.

**Figure 7:**
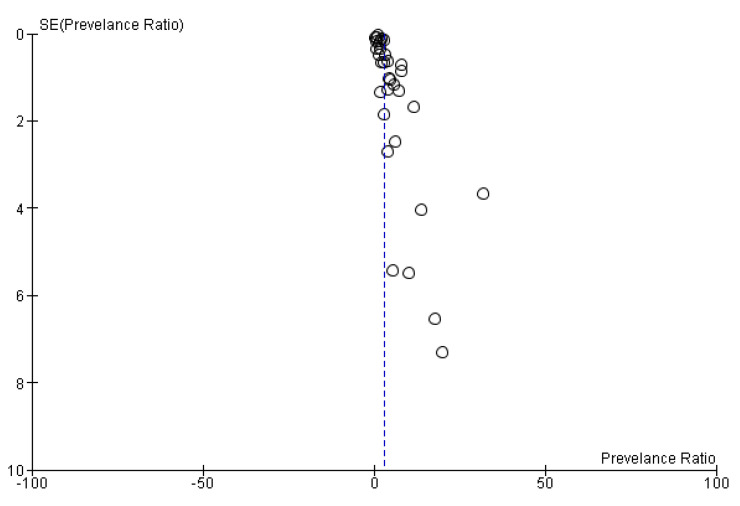
Funnel plot of publication bias.

The pooled prevalence was estimated at 3.05% (2.58–3.52, 95%CI), but with high heterogeneity (Ι^2^ 96%). The forest plot of the pooled prevalence is presented in Figure 6. Below is illustrated the forest plot and the funnel plot where the prevalence ratio and the standard error are distinguished, while random effects were used to estimate the pooled prevalence. Funnel plot is presented in Figure 7. 

#### Subgroup Analysis

##### Subgroup (1) According to Treatment

**Figure 8:**
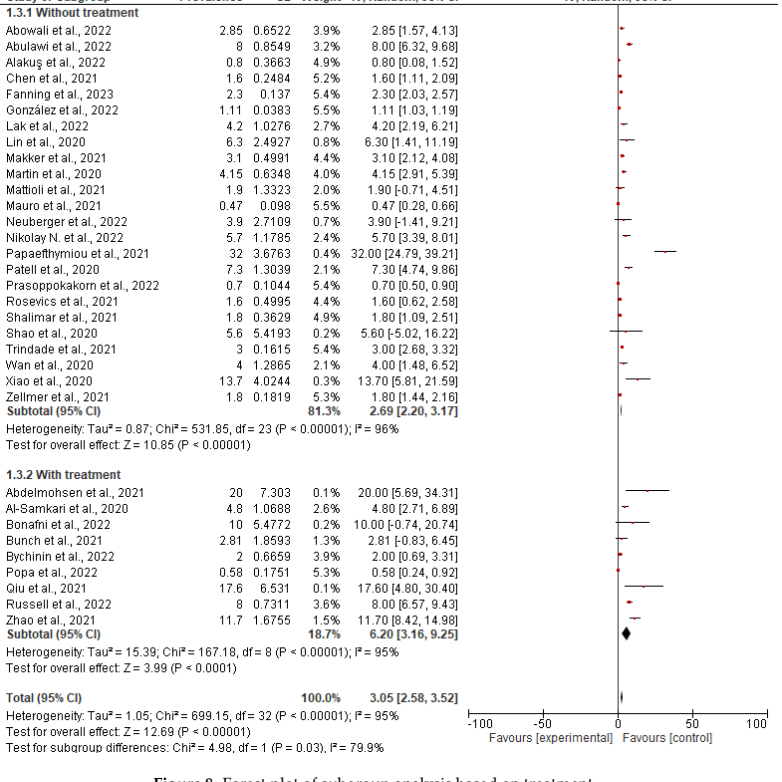
Forest plot of subgroup analysis based on treatment.

**Figure 9:**
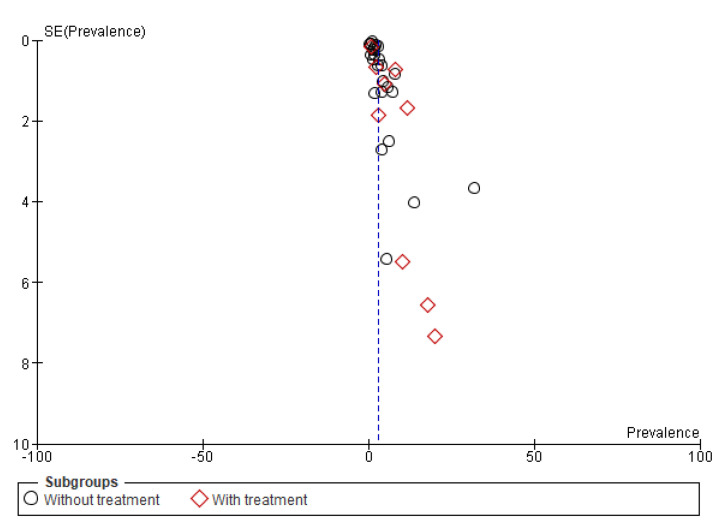
Funnel plot of publication bias on studies included in the subgroup (1) analysis.

Subgroup analysis was performed based on treatment. In 9 of the 33 studies in this systematic review, patients on anticoagulation or antiplatelet therapy were included. It seems that the use of anticoagulants or antiplatelet agents increases the risk of bleeding, with results also increasing the prevalence of gastrointestinal bleeding in these studies. However, even after removing these studies, the overall prevalence is estimated at 2.69% (2.20–3.17, 95%CI) quite close to the value of 3.05% which is in the total number of COVID-19 patients. The forest plot and the funnel plot show the prevalence distribution, the ratio, and the standard error. On the contrary, the total prevalence in these 9 studies is estimated at 6.2% (3.16–9.25, 95%CI). This value highlights the increased prevalence in patients on anticoagulation or antiplatelet therapy. Heterogeneity did not differ or was reduced in the subgroup analysis. Results are presented in Figure 8 and Figure 9. Despite this increased prevalence in anticoagulation-treated COVID-19 patients, a causal relationship between anticoagulants and gastrointestinal bleeding cannot be confirmed due to lack of data.

##### Subgroup (2) According to Location of Bleeding

**Figure 10:**
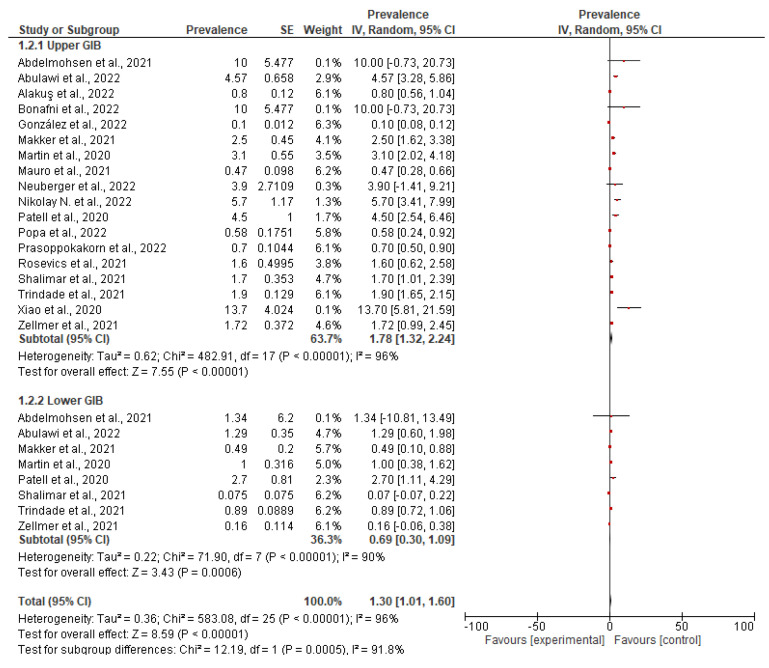
Forest plot of subgroup analysis according to the location of bleeding.

**Figure 11:**
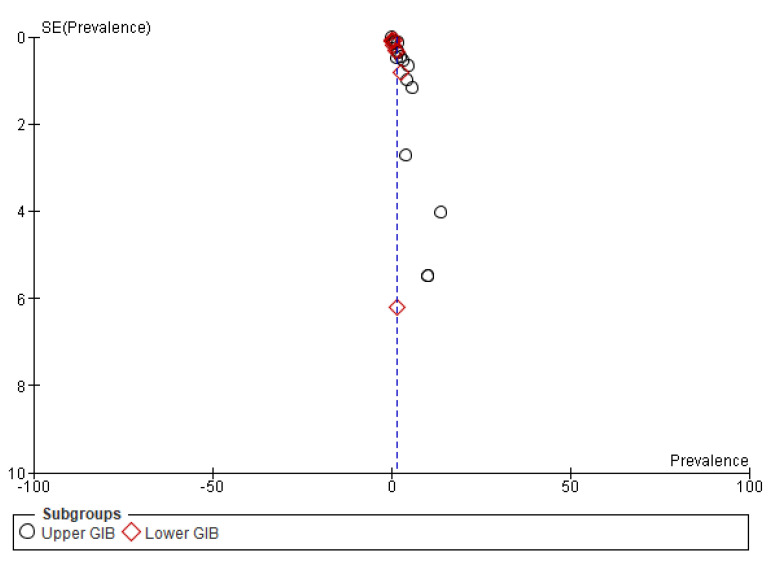
Funnel plot of publication bias on studies included in the subgroup (2) analysis.

In 17 of the 33 studies [26,27,28,29,30,31,34,35,37,38,43,44,48,53,54,55,56] in the meta-analysis, upper or lower gastrointestinal bleeding could be distinguished. Thus, it was possible to distinguish the prevalence of GI bleeding into the upper and lower gastrointestinal tract into two subgroups. A total of 15 studies reported the prevalence of upper gastrointestinal bleeding, resulting in a pooled prevalence equal to 1.78% [1.32, 2.24, 95% CI]. Accordingly, 8 studies report the prevalence of lower gastrointestinal bleeding and give a pooled prevalence equal to 0.69% [0.30, 1.09, 95% CI] or 1%. Thus, it is understood that upper gastrointestinal bleeding is a more common complication of COVID-19 than lower gastrointestinal bleeding, a fact that is also confirmed by the clinical findings. The results are visible in Figure 10 and Figure 11.

### 3.6. Publication Bias

Publication bias was performed with the construction of funnel plots which are presented in Figure 7 and Figure 9. The funnel plots showed asymmetry which is evidence for publication bias.

## 4. Discussion

In this systematic review, 33 studies were included that addressed the risk of GIB in COVID-19 patients. A total of 134,905 patients with COVID-19 participated in them, of which 1458 presented GIB. However, the prevalence of GIB differed between the studies, with the lowest value of 0.47% and the highest reaching 19%. The prevalence of GIB is influenced by several factors. It was found to be higher in studies involving COVID-19 patients on anticoagulation factors or in patients with gastrointestinal disturbances. In contrast, in studies involving patients with COVID-19 without signs of gastrointestinal involvement or in studies where the sample of patients was very large, the prevalence of GIB ranged up to 5%. Even this percentage, however, is quite significant, especially if the dangerousness of the situation is considered.

Our findings from the meta-analysis showed that the pooled prevalence of gastrointestinal bleeding is 3.05%. The 95% confidence interval of the prevalence ranges between 2.58 and 3.52, while the heterogeneity is high, reaching 96%. This percentage ranks gastrointestinal bleeding among the important complications of COVID-19, but not among the most common. However, due to the seriousness of this complication, it should be treated with caution. These percentages increase even more, reaching 6.2% (3.16–9.25, 95%CI) in patients receiving anticoagulant or antiplatelet treatment. This treatment is very common in COVID-19 patients due to the coagulopathy caused by the virus. Therefore, in patients under treatment, any symptom suggestive of gastrointestinal bleeding should be investigated, as well as gastrointestinal bleeding, and included in the differential diagnosis of various complications.

The guidelines suggest that patients presenting with GIB should undergo endoscopy within twenty-four hours, for diagnostics and therapeutic purposes [74]. However, COVID-19 patients are a different case as they are at greater risk due to pneumonia. Also, the intubation of many COVID-19 patients is another obstacle in performing endoscopy. Finally, the risk of transmission of SARS-CoV-2 increases during such operations [75]. For this reason, the endoscopic approach to patients must be conducted with great care as long as the conditions allow it. If endoscopy is not possible, then conservative treatment is preferred. This is based on taking proton pump inhibitors, and antibiotics, while this can be enhanced by taking somatostatin and terlipressin [76,77]. Finally, fresh frozen plasma (FFP), packed red blood cells, and intravenous fluid resuscitation are administered [38].

Of the 1458 cases, 286 underwent endoscopic procedures. Gastrointestinal endoscopic procedures include esophagogastroduodenoscopy, colonoscopy, and capsule endoscopy. Esophagogastroduodenoscopies were performed in the majority of those 286 cases, as in most cases the bleeding was in the upper gastrointestinal tract. Of the 286 endoscopies, 54 had a therapeutic purpose. The endoscopic treatment of bleeding includes various techniques; the most commonly performed are injectable solutions, hemostatic substances with local action, mechanical methods, elastic rings, hemostatic knots, stapling mechanisms, or cauterization. If these fail, surgical treatment is indicated. The success of these mechanisms depends on various factors such as age, hematemesis, oligemic shock, low hematocrit, as well as other concomitant diseases [78,79,80]. Of the injectable solutions, adrenaline is preferred. Clips are used on endoscopically visible bleeding vessels. Elastic rings are preferred in variceal bleeding, while hemostatic knots are preferred in polypectomy bleeding.

In the era of COVID-19, therefore, a decrease in endoscopies was observed [81,82]. However, in some cases it was deemed necessary to perform endoscopy to manage GIB. These cases mainly involved males, with an increased body mass index, anticoagulant treatment, and multiple comorbidities [83,84]. However, complications due to COVID-19 in these patients led to reduced 30-day survival, with an odds ratio of 0.25 [85]. Despite reduced survival, no increase in major rebleeding was found [85]. Taking all this into account, the risks of these interventions may outweigh the benefits [84]. In addition, cases have been reported in which GIB in COVID-19 patients was self-limiting without the need for any additional intervention [86]. Finally, increased intensive care unit stays and intubations were observed for COVID-19 cases undergoing endoscopies for the management of GIB [87].

Abnormalities in normal laboratory values are also seen in COVID-19 patients with gastrointestinal bleeding. The drop in hemoglobin is evident, reaching up to the value of 7.2 g/dL, while the normal value in males is between 13.5 and 17.5 g/dL, but in females is 11.5–15.5 g/dL. The platelets appear to be in the lower normal limits, and in some cases, they are slightly reduced. Normally they should be within the range of 140–400 × 10^3^/mm^3^. Prothrombin time and INR are indicators of the body’s coagulation capacity and are used as an initial control test to detect deficiencies of one or more coagulation factors (factors II, V, VII, X) as well as to monitor patients under anticoagulant therapy [60]. These seem slightly affected while finally the d-dimers (soluble fibrin degradation product) [61] were in the majority of cases above normal values. The changes in these values are due to a combination of bleeding, COVID-19, and the anticoagulant treatment that many of these patients are under.

Therefore, the outcomes of GIB in COVID-19 patients differ from non-COVID-19 patients. This difference is recorded in the stay in the intensive care units (ICU), in the use of corticosteroid drugs, intubation, as well as in the causes of death, as these mainly concern the respiratory due to the COVID-19 infection [88,89,90]. No differences are observed in hemoglobin, in the administration of blood and crystalloid solutions, and the rate of rebleeding. Also, the etiology of the bleeding and its clinical findings do not differ [89,90]. Finally, during the period of COVID-19, a significant decrease was observed in the number of patients coming to the hospital with GIB, which is attributed to their unwillingness to visit the hospital, due to COVID-19 [91,92]. In general, the causative agents of gastrointestinal bleeding differ slightly from the causes blamed for gastrointestinal bleeding in COVID-19 patients. In both cases, the main cause of upper digestive bleeding is peptic ulcer, followed by gastritis, duodenitis, varicocele, and esophagitis [93]. A common causative agent in non-COVID-19 patients of upper gastrointestinal bleeding is Mallory-Weis syndrome [93,94]. The etiology is more varied when it comes to bleeding from the lower gastrointestinal tract. In non-COVID-19 patients, bleeding is primarily due to diverticular disease, polyps, neoplasms, and inflammatory bowel disease [16].

Regarding mortality from GIB, 218 deaths were reported in these 33 studies involving COVID-19 patients with GIB. However, the majority of deaths is not due to the bleeding itself but due to the COVID-19 infection. About 10 were directly due to GIB. Chen et al. suggest that GIB is an independent prognostic indicator of the composite endpoint and death [25]. These findings are also supported by Popa et al [30]. Popa et al. also suggest that the administration of anticoagulants does not affect mortality. [30] Additionally, Patell et al. report an increase in mortality in patients with active cancer who suffer from COVID-19 and experience bleeding episodes [48].

An also frequently reported finding from these studies concerns the increased risk of GIB in critically and seriously ill COVID-19 patients [25,27,34,42,43,44,50,52,53,55]. In particular, the risk of bleeding increases in patients who require admission to an intensive care unit or are already in it, in patients on mechanical ventilation, and patients with severe COVID-19 pneumonia. Also, Zhao et al. state that in seriously ill patients the risk of hidden GIB increases, possibly due to stress-related mucosal disease (SRMD) [42]. This can be seen during gastroscopy and was observed in 2.6% of patients with GIB admitted to the ICU [95,96]. Neuberger et al. also observed in vivo that severe disease of COVID-19 may be associated with duodenal SARS-Cov-2 infection, severe duodenitis, and subsequent bleeding complications [55,97]. Laboratory findings indicating an increased risk of ICU admission are elevated D-dimers and ferritin and low platelet count [51,57]. Finally, Al-Samkari et al. state that in severe COVID-19 patients, bleeding is major, with a worse prognosis [45]. These data explain why Mauro et al., where only non-ICU patients participated, calculated the lowest prevalence of GIB in COVID-19 patients (0.47%) [27].

The most common cause of lower GIB was found to be a rectal ulcer. However, Martin et al. stress that intrarectal catheters are another cause of LGIB. These are catheters that reduce the risk of perianal skin breakdown, while at the same time reduce healthcare workers’ exposure and the risk of hospital-acquired infections. These made them especially valuable in the COVID-19 pandemic. However, necrosis caused by pressure ischemia increases the risk of GIB, and this should be seriously considered before their use [43,98,99,100].

Patients suffering from COVID-19 often present with hyper-inflammation [62]. This hyper-inflammation is treated with the use of corticosteroids. Corticosteroids may also help treat COVID-related acute respiratory distress syndrome (ARDS) [63,64,65]. Of the 33 clinical studies used in this systematic review, many cases of COVID-19 patients who developed GIB while receiving corticosteroids were reported. However, the most important treatment in hospitalized COVID-19 patients that appears to be associated with the occurrence of GIB is the administration of anticoagulants [66,67]. It has been found that disturbances in the balance of vasodilator and vasoconstrictor angiotensin as well as the cytokines of sepsis caused by COVID-19 result in coagulation disorders. Up to 50% of COVID-19 patients will develop coagulopathies. [66,67,68,69,70,71] The treatment of them is based on the administration of anticoagulants and mainly low molecular weight heparin. Venous thromboembolic episodes (VTE) are very common in COVID-19 patients and may coexist with bleeding. Prophylactic treatment for venous thromboembolic events is based on anticoagulant treatment, which appears to increase the risk of bleeding, mainly gastrointestinal [101]. Despite the higher prevalence of gastrointestinal bleeding in COVID-19 patients who are under anticoagulant treatment, their association cannot be confirmed due to the lack of data about the percentage use of anticoagulants in COVID-19 patients without GI bleeding. Finally, cases of administration of proton pump inhibitors, which are administered to patients with gastric ulcers at high risk of GIB, were reported. Proton pump inhibitors have been shown to contribute significantly to the prevention of bleeding in patients on anticoagulation or antiplatelet therapy [72,73]. These patient amounts are the ones reported, as not all studies reported the treatment given to the patients.

Demelo-Rodriguez et al. included only patients receiving standard, intermediate, or therapeutic doses of VTE prophylaxis. Despite this, it appeared that the prevalence of GIB was not affected by the dose of the received treatment of anticoagulation [57]. It has also been found that prophylactic heparin treatment in COVID-19 patients reduces the need for cardiorespiratory support as well as increased survival in non-severe patients. Unfortunately, these do not apply to seriously ill patients [102,103]. Norepinephrine and ventilatory support are associated with thrombotic events, the most common of which is deep vein thrombosis [52].

**Figure 12:**
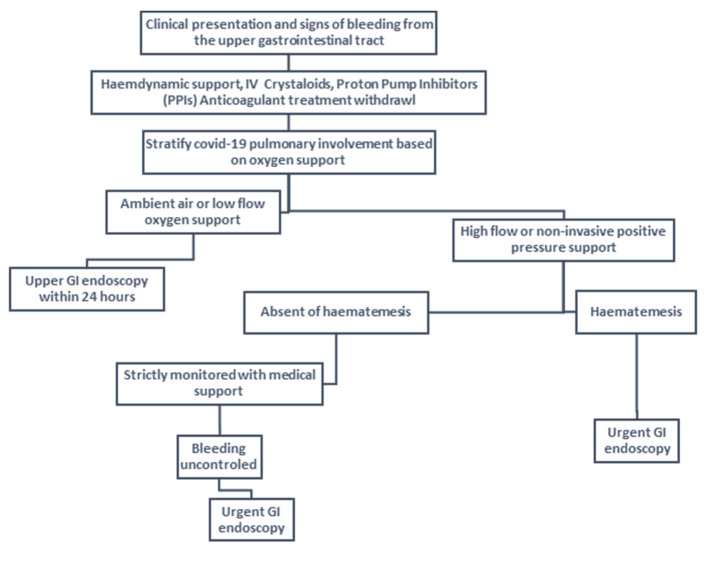
Management of upper gastrointestinal bleeding based on the algorithm proposed by Mauro et al. [27].

Mauro et al., proposed an algorithm (Figure 12) for the management of GIB in COVID-19 patients, especially upper. According to him, first, the hemodynamic status of the patient is controlled. Then, it is judged whether the patient needs respiratory support. Then, if the patient is at low risk, endoscopy can be performed to manage the bleeding within 24 h. Otherwise, in seriously ill patients, the degree of severity is judged according to the presence of hematemesis or not [27]. According to Wan et al., the presence of diarrhea may also indicate the severity of the infection and the need for non-mechanical ventilation [46,104]. Finally, imaging findings may also be useful in diagnosing GIB. In particular, in severe COVID-19 patients, it is considered very important to perform a CT scan when there are signs that indicate the existence of bleeding [53]. In Abdelmohsen et al., the most frequent indication for a CT scan was abdominal distension [54].

The meta-analysis of Marasco et al. estimated the overall prevalence of GIB in COVID-19 patients. This prevalence was estimated at 5%. The distribution of upper and lower bleeding was also calculated. In particular, 76.6% of the bleeding concerned the upper gastrointestinal system, while the rest concerned the lower [105]. This meta-analysis reviews only 10 observational studies and therefore only 91,887 cases of COVID-19. In contrast, the current systematic review included 33 studies and 134,905 cases of COVID-19.

Further research needs to be conducted on the safety of endoscopies in COVID-19 patients. It is also necessary to clarify how exactly GIB occurs in COVID-19 patients and whether it is caused directly by the infection or indirectly.

This meta-analysis has several strengths and limitations. Firstly, the updated literature search that includes newly published studies is the main strength compared to others. What is more, we followed a protocol for the literature search according to PRISMA guidelines. Furthermore, we conducted a subgroup analysis based on the presence of treatment or not. However, this research has some limitations. One of them is the absence of double-blind randomized studies, as only observational studies were found in the available literature. The small availability of studies on the subject, however, gives this systematic review strength, as it facilitates the synthesis of the results while simultaneously reducing the possibility of error.

## 5. Conclusions

In conclusion, COVID-19 patients show quite frequent gastrointestinal symptoms. Of them, GIB in COVID-19 patients is not the most common, as symptoms such as diarrhea, nausea, vomiting, and abdominal pain prevail. However, it occurs in a significant percentage and is one of the most important and dangerous complications in COVID-19 patients, where in some cases it requires urgent treatment and can even lead to death. Upper gastrointestinal bleeding is more common than lower, and the most common clinical findings are melena and hematemesis. Treatment and management of bleeding are based on either medication or endoscopic interventions such as embolization, while self-healing of bleeding is not uncommon.

## Figures and Tables

**Table 1:** PICO process.

P (patient/population)	General Population
I (intervention/exposure)	COVID-19 infection
C (comparison)	-
O (outcome)	Gastrointestinal Bleeding events/prevalence of GI bleeding

**Table 3:** Laboratory findings.

Study ID	Hematocrit (%)	Hemoglobin (g/dL)	Platelet (×10^3^/mm^3^)	Protomobin Time (s.)	International Normalized Ratio (INR)	D-Dimer (μg/mL)
Alakuş et al., 2022 [26]	22.1–33.8	7.2–11.2	88–192	13.3–15.2	1.16–1.3	1.8–2.3
Mauro et al., 2021 [27]	NM	9 (8.1–10.8)	NM	NM	NM	0.919 (0.621–2.046)
Trindade et al., 2021 [28]	NM	7.80 (6.80, 10.00)	NM	NM	NM	NM
Makker et al., 2021 [29]	NM	12 (±3)	236 (±143)	13	NM	1.034
Prasoppokakorn et al., 2022 [31]	22.5 ± 5.3	7.5 ± 1.8 (baseline: 12.6 ± 1.7)	NM	15.6 ± 5.8	1.41 ± 0.54	NM
González et al., 2022 [32]	NM	10.4 (3.2)	NM	NM	NM	NM
Zellmer et al., 2021 [35]	NM	In 22.2% of patients, it was <12	In 57.3% of patients, it was <200	NM	In 8.6% of patients, it was >1.25	NM
Abowali et al., 2022 [36]	NM	NM	NM	14.6 (13.5–16.8)	NM	0.905 (0.508–4.924)
Abulawi et al., 2022 [37]	NM	10.1 ± 2.2	NM	NM	NM	2.10 (1.17–10.16)
Shalimar et al., 2021 [38]	NM	7.2 (5.8–9.0)	90.5 (52–135)	NM	1.2 (1.2–1.4)	NM
Shao et al., 2020 [40]	NM	11.9 ± 3.2	177.50 ± 110.57	12.20 (11.50–13.40)	NM	0.49 (0.27–2.13)
Zhao et al., 2021 [42]	NM	12.6 (11.7–14.4)	161.0 (113.0–238.0)	14.2 (12.9–15.7)	NM	2.1 (0.9–11.4)
Martin et al., 2020 [43]	NM	7.5	250	NM	1.2	4.34
Al-Samkari et al., 2020 [45]	NM	NM	124 (95–154)	16.3 (14.6–17.4)	1.3 (1.2–1.4)	3.6(2.1–4.7)
Mattioli et al., 2021 [47]	NM	12.1 (10.9–13)	278.5 (186–348)	NM	1.25 (1.2–1.4)	1.4 (0.9–2.3)
Russell et al., 2022 [51]	NM	7.9 (6.7–8.6)	214 (155–290)	NM	1.1 (1.0–1.2)	1.7 (1.0–4.2)
Bychinin et al., 2022 [52]	NM	NM	189 (83.3–243)	NM	NM	0.98 (0.2–1.5)
Bonafni et al., 2022 [53]	NM	NM	239 (184–356)	NM	1.17 (1.08–1.49)	1.8 (1.1–3.1)
Demelo-Rodriguez et al., 2021 [57]	34% of patients were anemic	34% of patients were anemic	In 6% of patients, it was <100,000	In 32.5% of patients, it was >13.5 s	NM	In 94% of patients, it was >upper normal limit

NM: Not mention.

**Table 4:** Treatment of COVID-19 patients who experienced GIB.

COVID-19 Treatments during Hospitalization in Patients with GIB	
Corticosteroids	112
Anticoagulant and antiplatelet therapy	215
LMWH	110
PPI	141

LMWH: Low Molecular Weight Heparin, PPI: Proton Pump Inhibitors.

**Table 5:** Quality Assessment.

Study ID	Selection				Comparability	Outcome			Total	Quality
	Representativeness of the Exposed Cohort	Selection of the Non-Exposed Cohort	Ascertainment of Exposure	Demonstration that Outcome of Interest was not Present at the Start of the Study	Comparability of Cohorts on the Basis of the Design or Analysis Controlled for Confounders	Assessment of Outcome	Was Follow-Up Long enough for Outcomes to Occur	Adequacy of Follow-Up of Cohorts		
Abdelmohsen et al., 2021 [54]	*		*	*	**	*	*	*	8/9	GOOD
Abowali et al., 2022 [36]	*		*	*	**	*	*	*	8/9	GOOD
Abulawi et al., 2022 [37]	*		*	*	**	*	*	*	8/9	GOOD
Alakuş et al., 2022 [26]	*		*	*	**	*	*	*	8/9	GOOD
Al-Samkari et al., 2020 [45]	*		*	*	**	*	*	*	8/9	GOOD
Bonafni et al., 2022 [53]	*		*	*	**	*	*	*	8/9	GOOD
Bunch et al., 2021 [49]	*		*	*	**	*	*	*	8/9	GOOD
Bychinin et al., 2022 [52]	*		*	*	**	*	*	*	8/9	GOOD
Chen et al., 2021 [25]	*		*	*	**	*	*	*	8/9	GOOD
Fanning et al., 2023 [41]	*		*	*	**	*	*	*	8/9	GOOD
González et al., 2022 [32]	*	*	*	*	**	*	*	*	9/9	GOOD
Lak et al., 2022 [33]	*		*	*	**	*	*	*	8/9	GOOD
Lin et al., 2020 [39]	*		*	*	**	*	*	*	8/9	GOOD
Makker et al., 2021 [29]	*		*	*	**	*	*	*	8/9	GOOD
Martin et al., 2020 [43]	*		*	*	**	*	*	*	8/9	GOOD
Mattioli et al., 2021 [47]	*	*	*	*	**	*	*	*	9/9	GOOD
Mauro et al., 2021 [27]	*		*	*	**	*	*	*	8/9	GOOD
Neuberger et al., 2022 [55]	*		*	*	**	*	*	*	8/9	GOOD
Nikolay N. et al., 2022 [56]			*	*	**	*	*	*	7/9	GOOD
Patell et al., 2020 [48]			*	*	**	*	*	*	8/9	GOOD
Popa et al., 2022 [30]	*	*	*	*	**	*	*	*	9/9	GOOD
Prasoppokakorn et al., 2022 [31]	*		*	*	**	*	*	*	8/9	GOOD
Qiu et al., 2021 [50]	*	*	*	*	**	*	*	*	9/9	GOOD
Rosevics et al., 2021 [34]	*		*	*	**	*	*	*	8/9	GOOD
Russell et al., 2022 [51]	*		*	*	**	*	*	*	8/9	GOOD
Shalimar et al., 2021 [38]	*		*	*	**	*	*	*	8/9	GOOD
Shao et al., 2020 [40]			*	*	**	*			5/9	FAIR*
Trindade et al., 2021 [28]	*		*	*	**	*	*	*	8/9	GOOD
Wan et al., 2020 [46]	*		*	*	**	*	*	*	8/9	GOOD
Xiao et al., 2020 [44]	*		*	*	**	*	*	*	8/9	GOOD
Zellmer et al., 2021 [35]	*		*	*	**	*	*	*	8/9	GOOD
Zhao et al., 2021 [42]	*		*	*	**	*	*	*	8/9	GOOD

## Data Availability

No new data are created.
